# Investigation of Electrical and Wearing Properties of Wool Fabric Coated with PEDOT:PSS

**DOI:** 10.3390/polym15112539

**Published:** 2023-05-31

**Authors:** Julija Pupeikė, Audronė Sankauskaitė, Sandra Varnaitė-Žuravliova, Vitalija Rubežienė, Aušra Abraitienė

**Affiliations:** Center for Physical Sciences and Technology, Department of Textiles Technology, Demokratu˛ Str. 53, LT-48485 Kaunas, Lithuania; audrone.sankauskaite@ftmc.lt (A.S.); sandra.varnaite.zuravliova@ftmc.lt (S.V.-Ž.); vitalija.rubeziene@ftmc.lt (V.R.); ti@ftmc.lt (A.A.)

**Keywords:** conductive textiles, PEDOT:PSS, wool fabric, coated textile, electrical properties, wearing properties

## Abstract

The way to improve the properties (resistance to washing, delamination, and rubbing off) of the PEDOT:PSS coating applied on wool fabric without reduction of its electrical conductivity by introducing a commercially available combination of low formaldehyde content melamine resins into the printing paste is presented in this paper. Primarily, to improve the hydrophilicity and dyeability of wool fabric, the samples were modified using low-pressure nitrogen (N_2_) gas plasma. Two commercially available PEDOT:PSS dispersions were used to treat wool fabric by the exhaust dyeing and screen printing methods, respectively. Spectrophotometric measurements of the color difference (ΔE*_ab_) and visual evaluation of woolen fabric dyed and printed with PEDOT:PSS in different shades of the blue color showed that the sample modified with N_2_ plasma obtained a more intense color compared to the unmodified one. SEM was used to examine the surface morphology and a cross-sectional view of wool fabric that had undergone various modifications. SEM image shows that the dye penetrates deeper into the wool fabric after plasma modification using dyeing and coating methods with a PEDOT:PSS polymer. In addition, with a Tubicoat fixing agent, HT coating looks more homogeneous and uniform. The chemical structure spectra of wool fabrics coated with PEDOT:PSS were investigated using FTIR-ATR characterization. The influence of melamine formaldehyde resins on the electrical properties, resistance to washing, and mechanical effects of PEDOT:PSS treated wool fabric was also evaluated. The resistivity measurement of the samples containing melamine-formaldehyde resins as an additive did not show a significant decrease in electrical conductivity, while the electrical conductivity was maintained after the washing and rubbing test as well. The best results of electrical conductivity for investigated wool fabrics before and after washing and mechanical action were determined for samples subjected to the combined processing–surface modification by low-pressure N_2_ plasma, dyeing by exhaust with PEDOT:PSS, and coating by the screen-printing method of PEDOT:PSS and a 3 wt.% melamine formaldehyde resins mixture.

## 1. Introduction

**Figure 1:**
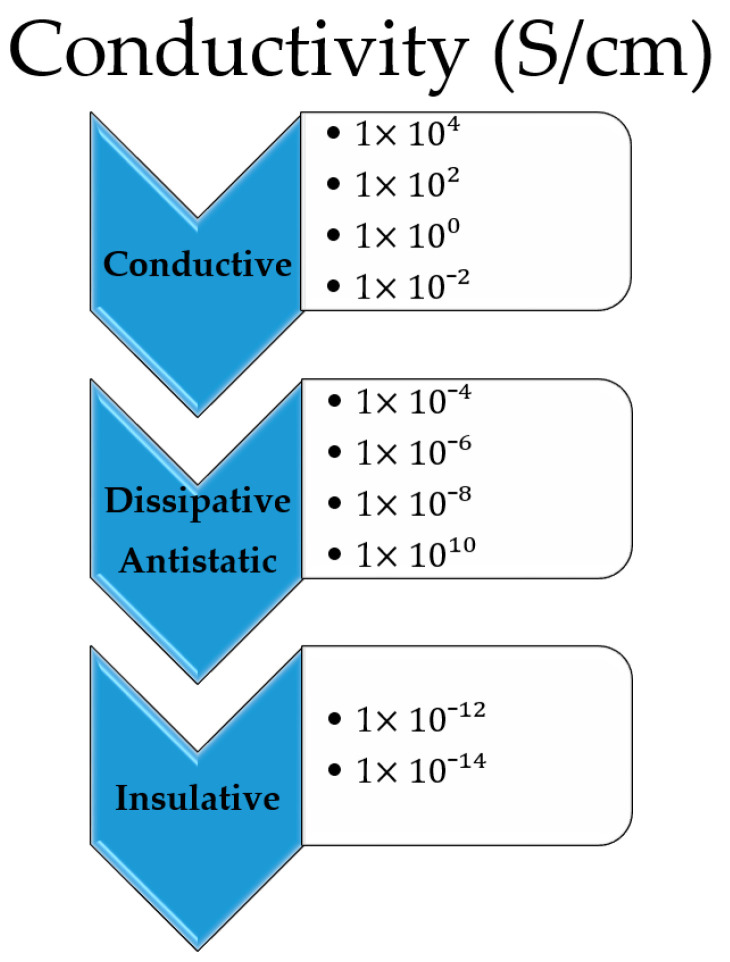
Conductivity ranges for different applications [17,21].

The next major advancement in technology in the contemporary period will likely be wearable electronics and conductive materials, which will surpass portable devices. Conductive fabrics can be utilized to incorporate sensors capable of sensing and responding to external stimuli [1,2,3,4,5]. The stimuli and responses can have a variety of causes, including chemical, thermal, magnetic, and electrical [1]. The development of lightweight, flexible parts and fibrous structures with a high electrical conductivity that can withstand the stresses of wearing, and caring for the textile is a critical obstacle to the success of wearable e-textile technology [2,6]. In fact, there are several obstacles to be overcome in the quickly expanding field of electronic textiles, or e-textiles, due to the rigidity and weight of metallic conductors [2,7]. Electrostatic discharge terminology glossary (ESD ADV1.0) defines conductive materials as “a material that has a surface resistance of less than 1.0 × 10^4^ Ω or volume resistance of less than 1.0 × 10^4^ Ω”. In order to combine electronic devices, such as sensors [8,9,10,11,12], antennas [7,13], and energy storage devices [14,15,16], new technology has been used in textile construction. Electrostatic discharge (ESD) is a very common and easily attainable phenomenon in the realm of alternative nonmetal-based conductive fabrics [17,18,19,20]. Components for smart textiles need to have a conductivity that is significantly higher than the electrostatic discharge range to replace metallic conductors in fabrics that effectively transmit information and support computing. According to the ESD Association standard [21], dissipative materials have conductivities between 1 × 10^−11^ S/cm and 1× 10^−4^ S/cm. Contrarily, conductive materials are those that have conductivity greater than 1 × 10^−4^ S/cm (Figure 1). To create lightweight materials that potentially replace metallic fabrics, higher conductivity must be combined with good mechanical qualities [21].

Conductive textiles can be made using natural and chemical fibers with conductive additives, such as conductive polymers, metals, oxides, and carbon. Intrinsically conductive polymers (ICPs), such as polyaniline (PANI), polypyrrole (PPy), and poly(3,4-ethylenedioxythiophene)-poly(styrenesulfonate) (PEDOT:PSS) can be used to create conductive coatings or tracks on textiles [22]. These ICPs can be applied to textiles through conventional methods, such as exhaust dyeing, coating, or screen printing, and may be fixed with a binder or directly reacted with the functional groups of the fibers [23,24,25,26,27,28]. When creating electrically conductive textiles, it is crucial to take into account the characteristics of wearing comfort, breathability, flexibility, softness, resilience to repeated washings, and mechanical elements during daily use. In this study, wool fabric was modified with conductive polymer PEDOT:PSS with low-formaldehyde melamine resins to create not only conductive but also flexible and wearable textile. Wool is a natural protein fiber composed of 18 amino acids with amino and carboxy groups and sulfur, linking adjacent macromolecules by cross-disulfide bonds. Wool fibers are usually dyed with acid dyes. Because of the PSS negatively charged sulfonate ions (-SO_3_-) interactions with the cationic amine groups of the wool fibers, PEDOT:PSS is referred to as a “conducting acid dye” [25,29] that may attach to protein fibers and dye them in various shades of blue [27]. There are several advantages for the PEDOT:PSS water-soluble polyelectrolyte system for textile applications, including electrical conductivity, applicability with traditional textile technologies, commercial availability, water solubility, stability, and high visible light transmittance [30]. A disadvantage influenced by the solubility of the conductive polymer component polystyrene sulfonate (PSS) in water causes the formed coating to peel off from the textile, thus decreasing its resistance [22,23,29] to wet treatments. It is also important to evaluate the ability of the PEDOT:PSS coating on wool fabric to retain its conductivity and resistance to rubbing and washing [31,32,33,34,35]. Physical and chemical testing, as well as color change measurement, can help to determine the durability of PEDOT:PSS coating on textile materials under different processing conditions and inform about the most appropriate treatment with this conductive polymer [31,36]. Modification, etching, activation, cleaning and hydrophilicity of the wool fiber surface were performed using low-pressure nitrogen (N_2_) gas plasma [37,38,39,40]. Scanning electron microscopy (SEM) was used to analyze the surface morphology, defects, contaminants, and roughness of textile materials. It helps to characterize the microstructure of fibers and yarns, as well as the surface texture and finish of textiles [34,35,40,41]. FTIR-ATR spectroscopy was used to identify the chemical bonds present in the sample and to determine the chemical composition of the investigated sample [37,42,43,44,45].

The novelty of this work is to design and characterize electrically conductive textiles coated with a formulation containing a water dispersion of PEDOT:PSS as a conductive additive and increased resistance to mechanical and wet effects such as dry rubbing and washing.

## 2. Materials and Methods

**Figure 2:**
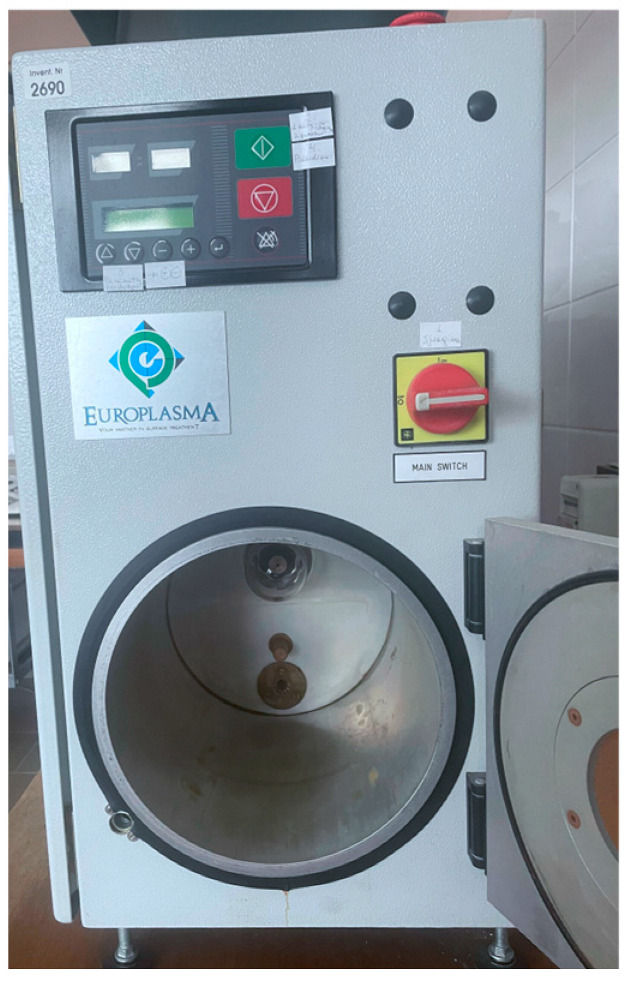
Junior Plasma System 004/123 (Europlasma, Oudenaarde, Belgium).

A 100% wool fabric used as the substrate for this research was acquired from JSC “Drobė” (Kaunas, Lithuania). The Junior Plasma System 004/123 (Europlasma, Oudenaarde, Belgium) is a low-pressure plasma apparatus that was used to modify the wool fabric samples with N_2_ gas. The chosen process parameters were as follows: processing duration of 3 min, pressure of 0.4 mbar, discharge power of 200 W, and N_2_ gas flow of 0.01 L/min (Figure 2).

**Figure 3:**
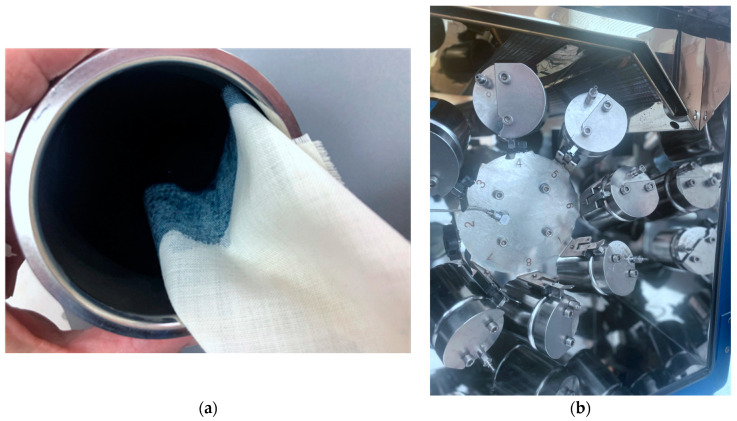
Wool fabric sample immersed into PEDOT:PSS water dispersion (**a**), views of cups in laboratory dyeing apparatus, Ahiba Nuance ECO (**b**).

The PEDOT:PSS commercial products Clevios F ET and Clevisios SV3 (Heraeus Holding GmbH, Hanau, Germany) were chosen because of their suitable chemical composition and viscosity for our dyeing/coating methods. The PEDOT:PSS with ethylenediol (5–10%) commercial additive water dispersion Clevios F ET was used to dye wool fabric samples that had not been treated, or that had been treated with N_2_ plasma (13 cm × 20 cm) in various shades of blue using the exhaust dyeing method (Figure 3a). Using Clevios F ET at various pH levels, dyeing was performed at 90 °C for 30 min. After dyeing, samples were dried in a lab drying-thermosetting machine called the TFOS IM 350 (Roaches International, Batley, UK) for three minutes at 100 °C. The Clevios F ET dyed and undyed wool fabrics samples were printed using the flat screen-printing method with the commercial PEDOT:PSS water dispersion Clevios S V3 (Heraeus Holding GmbH, Hanau, Germany) and dried at 100 °C for 3 min in a drying–thermosetting machine TFOS IM 350. Cross-linking can be used to stabilize films and improve delamination on the fabrics. However, some of them, such as glycidoxy propyltrimethoxysilane (GOPS), require high curing temperatures (140 °C for 1 h) and have an adverse impact on conductivity values [46]. Our research has shown that wool fabric turns yellow at temperatures above 105 °C. Melamine formaldehyde resin systems are used for textiles to produce highly durable coatings, especially for impregnation against moisture [41]. For this reason, some of the investigated samples were treated with a low formaldehyde content melamine resin named Tubicoat fixing agent HT (CHT Germany GmbH, Tübingen), which does not require high curing temperatures and can improve the properties of PEDOT:PSS coatings on wool fabric, such as washing resistance, delamination resistance, and mechanical resistance, without compromising it electrical conductivity [41,43,44].

Clevios S V3 and 3 wt.% Tubicoat fixing agent HT were used to screen print the wool fabric samples for this purpose. The samples were then dried at 100 °C for 5 min in a lab drying–thermosetting machine called the TFOS IM 350. The codification of samples treated using different methods is shown in Table 1.

### 2.1. SEM Analysis

SEM microscopy and FTIR-ATR (attenuated total reflection) spectroscopy have been used to study the surface of fully coated samples. With scanning electron microscopy (SEM), Quanta 200 FEG (FEI, Eindhoven, The Netherlands), at 20 keV (low vacuum), magnifications of 500× and 5000× work distances of 6.0 mm, low vacuum pressures of 80 Pa, and large-field detectors (LFD), the surface morphology of the coated wool fabric was examined.

### 2.2. Fourier Transform Infrared Spectroscopy with Attenuated Total Internal Reflectance (FTIR-ATR)

Utilizing FTIR-ATR spectroscopy (FTIR Perkin Elmer Frontier, MA, USA), chemical bonding analyses of untreated and plasma-treated wool samples dyed with Clevios F ET and coated with Clevios SV 3 were carried out. By analyzing the infrared spectra of the tested samples, which were measured using FTIR-ATR spectroscopy in ATR reflection mode (spectrum range: 600–4000 cm^−1^, resolution: 1 cm), it was possible to investigate the structural modifications that had taken place in the samples.

### 2.3. Evaluation of the Durability of Conductive PEDOT:PSS Coating after Washing

Each without and with N_2_ plasma-modification samples after dyeing with Clevios F ET and coating with Clevios SV 3 (see Table 1) were washed and dried 5 cycles. The method used for the five wash cycles according to ISO 13688:2013 [47]. Samples were washed with Scourotester Computex (Budapest, Hungary), washing temperature 40 °C (method A1S) and duration 30 min (ISO EN 105-C06) [48]. Washed wool fabrics were line dried in ambient atmosphere. 

### 2.4. Color Fastness to Rubbing in the Conditioning Atmosphere

A testing instrument with two different sizes of rubbing fingers and a reciprocating straight rubbing action was used to determine the color fastness against dry rubbing. The specimen and rubbing cloth were subjected to a standard environment of (20 ± 2) °C and (65 ± 4)% for at least 4 h prior to testing [49]. Each specimen was secured to the testing device’s base such that its long axis would align with the device’s track. To minimize specimen movement, a space was established between the baseplate of the test fixture and the specimen and between the baseboard of the testing device and the specimen. When laid flat on the tip of the finger, the weaving of a conditioned rubbing cloth is parallel to the direction of finger rubbing. On the dry specimen, a track measuring 104 ± 3 mm in length was rubbed 20 times in a straight line, 10 times forward and 10 times back, at a rate of one cycle per second, with a downward force of 9 ± 0.2 N [50]. Following testing, the staining of the rubbing cloth was evaluated using a grey scale for staining in proper lighting [51].

### 2.5. Evaluation of Color Change

The color change of dyed and coated samples after washing was evaluated on a grey scale. Tests for color fastness were performed according to the grey scale for determining changes in the color of textiles. Pairs of matte-finish gray color chips (or samples of matte-finish grey fabric) make up the basic scale, which has five stages. An enhanced scale also has four half-steps, making a total of 9 steps. The standard offers a precise colorimetric scale definition that can be used to compare samples that may have altered with newly prepared working referents [52].

### 2.6. Color Intensity Measurements

**Figure 4:**
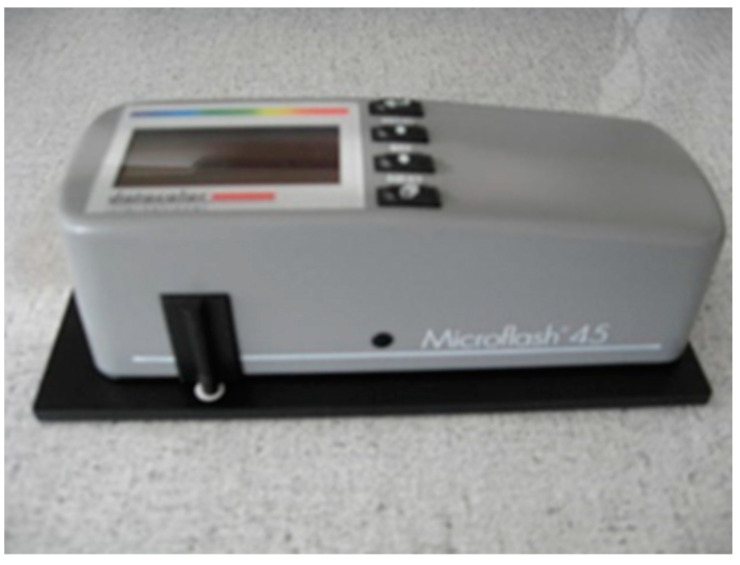
Spectrophotometer Datacolor Microflash MF 45 IR.

The color difference (ΔE*ab) of the unmodified and modified plasma, Clevios F ET dyed and Clevios S V3 printed samples (Table 1) after washing and rubbing tests were evaluated with Datacolor Microflash MF 45 IR spectrophotometer (Datacolor AG, Rotkreus, Switzerland) [53] (Figure 4). The wavelength range of the apparatus was: visible range 400–700 nm and infrared range 700–1100 nm, geometry type 0°/45°, illuminant and observer conditions D_65_/10°, area of view 3.2 mm, number of readings per sample—4 and number of measured layers of fabric—4. The standard atmosphere temperature (20 ± 2) °C and relative humidity (65 ± 4)% were used to keep the samples prior to testing [49]. The standard deviation was roughly 1, and measurements were done on 5 elementary samples.

The average of 4 readings per sample was recorded, and the standard deviation was approx. ±1σ, namely, color yield (K/S), the substrate absorption function (K), the scattering function of background (S), and the reflectance (R) in the visible spectrum (400–700 nm). Other color parameters, such as L*, indicate the difference between darkness (where L* = 0) and lightness (where L* = 100); C* represents the saturation/chroma of the color; a*, the red/green coordinate is the difference between red (+a*) and green (−a*); h is the hue angle (h sample minus h standard), which is the difference in hue; b* is the yellow/blue coordinate, which is the difference between blue (−b*) and yellow (+b*).

### 2.7. Evaluation of Electrical Properties

**Figure 5:**
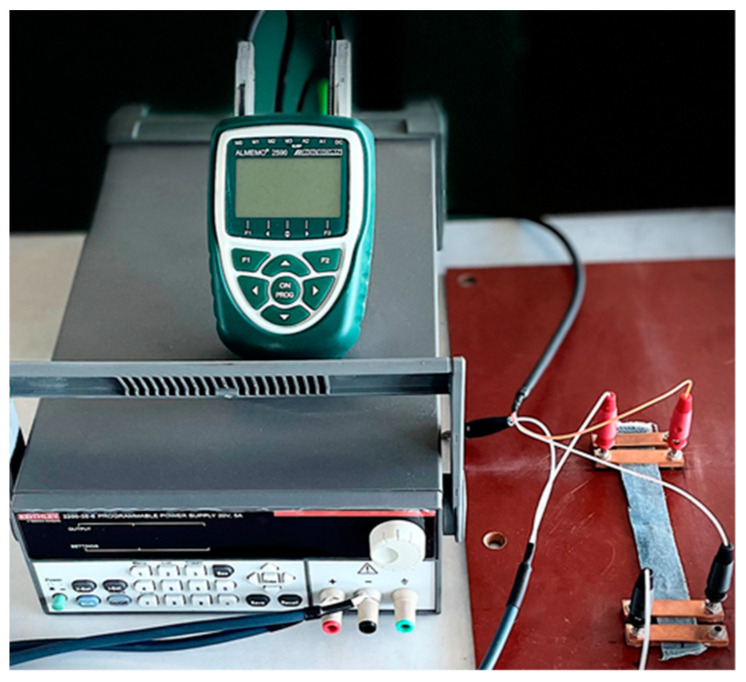
Electrical resistance measurement. The electrodes were positioned and covered the entire width of the conductive track, as illustrated in the image.

**Figure 6:**
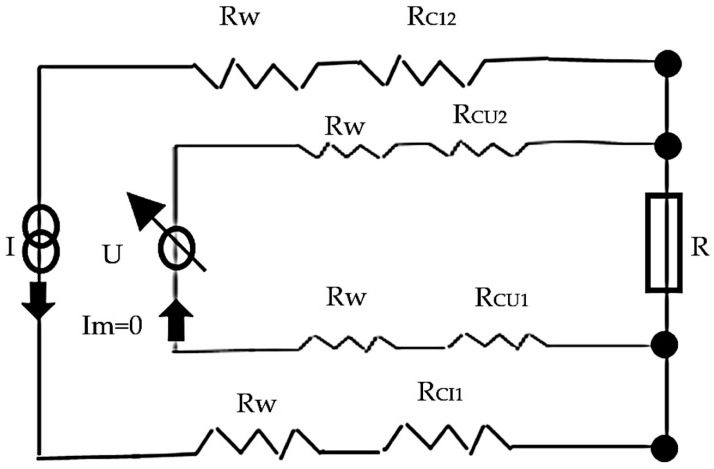
Detailed scheme for the “four electrode−four wire method”; the four electrodes (contacts) are visualized by the four nodes indicated in the scheme.

Electrical resistance measurements, which are the inverse of electrical conductivity measurements, were used to identify all fabrics created for this study. Before testing, the specimens were left for at least 24 h in standard atmosphere conditions (20 °C and 65% RH) [49]. The voltage measurements were provided in the same conditions. The linear resistance was measured six times in 1 min, every 10 s, and the distance between the voltage electrodes is (d) 10 cm, and a sample width of 2 cm (Figure 5). Measurements were made according to the Four Point Kelvin method [54]. The electric current (I) is 0.01 ÷ 0.001 A, the resulting voltage (U) and the sample resistance (R) is calculated according to Formula (2), and the linear resistance (RL) is calculated according to Formulas (1 and 3) (Figure 6).

I is the applied current in amperes; U is the measured voltage in volts, and I_m_ is the current in the voltage measurement circuit (equivalent to zero)

(1) $$R\;\mathrm{the}\;\mathrm{resistance}\;\mathrm{of}\;\mathrm{sample}\;(\mathrm{function}\;\mathrm{of}\;\mathrm{electrode}\;\mathrm{spacing}\;d)=R_{L}\times d,\;\mathrm{in}\;\mathrm{ohm}$$

R_CI1_ and R_CI2_ are the contact resistances in the current circuit in ohms; R_CU1_ and RCU2 are the contact resistance in the voltage circuit in ohms; RW is the wire resistance in ohms.

R_CI_, R_CV_, and R_W_ can be excluded due to the ”four electrode−four wire measurement” so that the resistance of the specimen can be calculated by the simple formula

R = U/I (2)

The linear resistance R_L_, in ohm/m, is then calculated as

R_L_ = R/d (3)

The distance d between the voltage electrodes is 10 cm.

## 3. Results

### 3.1. SEM Analysis

**Figure 7:**
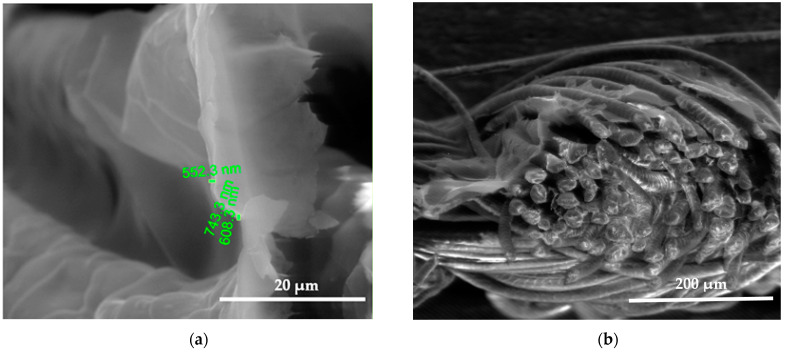
SEM images of the wool fiber cross-section sample (S) with Clevios S V3 coating applied by the printing method. Magnification 5000× (**a**) and 500× (**b**).

**Figure 8:**
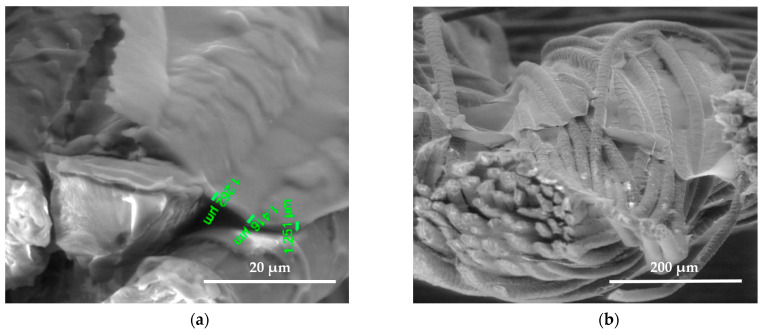
SEM images of the wool fiber cross-section sample (PS) N_2_ plasma-treated and Clevios S V3 screen printing method coating; magnification 5000× (**a**) and 500× (**b**).

**Figure 9:**
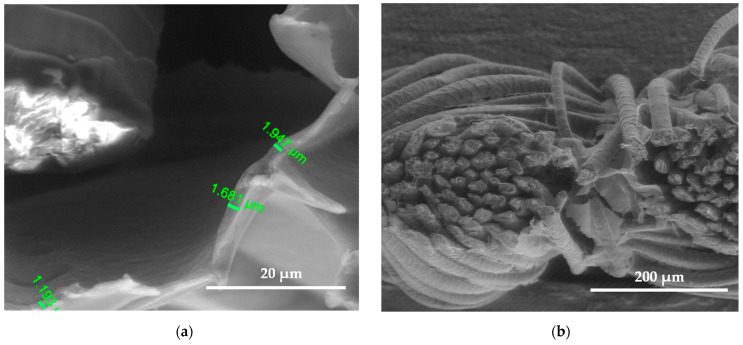
SEM images of the wool fiber cross-section sample (PFS) N_2_ plasma-treated Clevios F ET dyed and Clevios S V3 screen printing method coating; magnification 5000× (**a**) and 500× (**b**).

**Figure 10:**
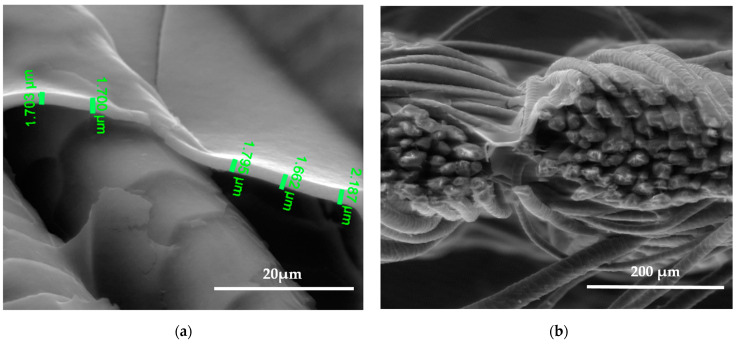
SEM images of the wool fiber cross-section sample (PFSH) N_2_ plasma-treated Clevios F ET dyed and Clevios S V3 with Tubicoat fixing agent HT screen printing method coating; magnification 5000× (**a**) and 500× (**b**).

SEM images show the differences between unmodified and modified plasma wool samples with different coating combinations, according to Table 1. SEM images show that when comparing the samples without (S) and after plasma modification (PS, PFS), the thickness of the sample (S) coating is thinner at 0.552 ÷ 0.743 µm, uneven, and delaminated (Figure 7a,b), but after plasma modification of the sample (PS) we obtain a thicker coating 1.251 ÷ 1.416 µm, the sample is ignited more evenly, and the spaces between the threads are filled with a coating (Figure 8a,b) [37]. After plasma modification of samples (PFS) (Figure 9a,b), the coating is thicker at 1.193 ÷ 1.947 µm and more equal and well penetrated on the other side of the fabric in comparison to samples (PS) [38,39,40] without initial dyeing, the coating is only formed and visible on the surface (Figure 8b). Comparing samples without (S, PS, PFS) and with Tubicoat fixing agent HT (FSH), the coating thickness is 1.662 ÷ 2.187 µm (Figure 10a,b) and looks more homogeneous and uniform than without Tubicoat fixing agent HT (Figure 7, Figure 8 and Figure 9).

### 3.2. Evaluation of Conductive PEDOT: PSS Coating Durability after Washing Cycles

The photo images of plasma and PEDOT:PSS treated wool samples (F, PF, S, PS, FS, PFS, SH, FSH, PFSH) before and after five washing and drying cycles are present in Table 2. The color change analysis showed that the samples after plasma treatment are more than half-steps [52] when compared to other samples: F with PF, S with PS, and FSH with PFSH. This indicates that the plasma-treated samples became darker, possibly due to better absorption of PEDOT:PSS. After five washes, the plasma-treated samples (PF, PS, PFS, PSH) showed less color change compared to the untreated samples (F, S, FS, SH). Plasma treatment cleaned the surface of the wool fabric, and we assume that it increased the yield of absorbed PEDOT:PSS and resistance during washing as well [37,38,39,40]. When we compared unwashed with sample after five washes cycles (F, PF, S, PS, PFS), according to gray scale a color change was found about one stages or more [52]. Furthermore, comparing PEDOT: PSS coated samples with (SH, FSH, PFSH) and without Tubicoat fixing agent HT (S, FS, PFS) before and after five washes, the color change between washed and unwashed samples was less than half-steps according to grey scale. The received results showed that Tubicoat fixing agent HT increased the resistance to washing. The sample PFSH demonstrated the highest wash fastness and the lowest color changes after five washes (Table 2).

### 3.3. Color Fastness to Dry Rubbing Analysis

Improved resistance to dry rubbing was obtained by using the same combination of with or without plasma modification, dyeing, and dyeing/coating with PEDOT:PSS with or without Tubicoat fixing agent HT. After dyeing with Clevios F ET, the color of the sample (F) was so dull that it was difficult to evaluate the rubbing test result, but it became more visible after plasma treatment (PF) (Table 3). Better dry rubbing resistance of samples (SH, PSH, FSH, PFSH) was obtained when 3 wt.% of Tubicoat fixing agent HT was added to the Clevios S V3 (Table 4). The tests carried out showed that the best resistance of rubbing was obtained with the combined modification (PFSH): plasma treatment, PEDOT:PSS dyeing and coating, and Tubicoat fixing agent HT adding (Table 4).

### 3.4. Measurements of Electrical Resistance

**Figure 11:**
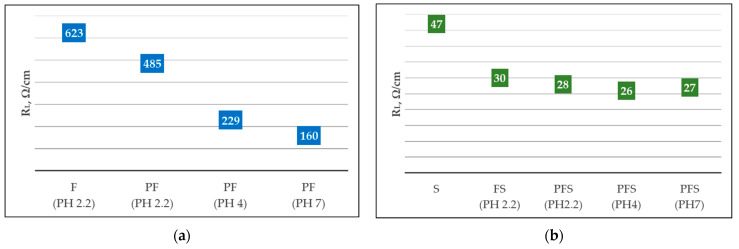
Measurements of the linear resistance (R_L_) of untreated and plasma-treated wool fabrics at different pH values of Clevios F ET (**a**), Clevios S V3, and Clevios F ET/Clevios S V3 (**b**).

Comparison of plasma (160 Ω/cm) and no plasma (623 Ω/cm) treatments of wool fabrics (Figure 11a) resulted in an approximately 4 times lower linear resistance (R_L_). The pH change (2.2, 4, and 7) dyeing with Clevios F ET alone resulted in the lowest resistivity at pH 7 (160 Ω/cm) and was not influence the resistivity of newly formed coating with Cevios S V3 (Figure 11b). Plasma treatment, dyeing, and coating with PEDOT:PSS (sample PFS) resulted in almost a factor of two times lower linear resistivity measurements (26 ÷ 28 Ω/cm) compared with only coating Clevios SV 3 (sample S) (47 Ω/cm) (Figure 11a,b). Since changing the pH did not significantly affect the linear resistance reduction, we did not change the pH in further tests and used the original Clevios F ET.

**Figure 12:**
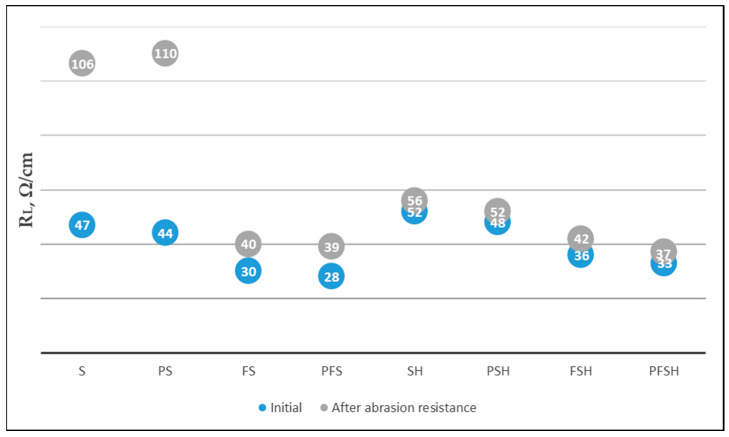
Measurements of linear resistance (R_L_) untreated and after plasma treatment with different modifications of wool fabrics after rubbing testing.

**Figure 13:**
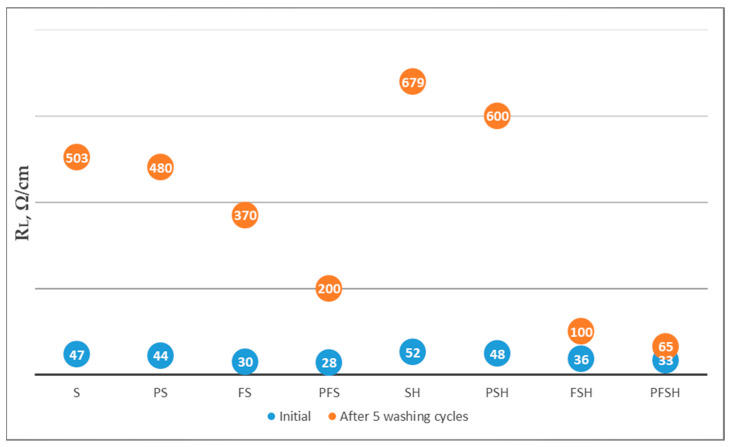
Linear resistance (R_L_) measurements on untreated and plasma-treated wool fabrics coated with different modifications of Clevios F ET and Clevios S V3 after five washing cycles.

The linear resistance (R_L_) of all samples with the same chemical modifications with PEDOT:PSS after plasma treatment (PS, PFS, PSH, PFSH) were twice as low as without plasma treatment (S, FS, SH, FSH) (Figure 12). This is due to the better affinity and binding of PEDOT:PSS as a “conductive acid dye” [25] to the cationic amine sites of the wool fiber after plasma treatment. Samples with Tubicoat fixing agent HT (SH, PSH, PFSH) retained better electrical conductivity after washing and rubbing compared to those without Tubicoat fixing agent HT (S, PS, FS, PFS) (Figure 12). It is assumed that Tubicoat fixing agent HT as a cross-linker [55] affected the formed fabric surface film resistance to rubbing; through this, the resistance (33 Ω/cm) did not increase (Figure 12). After rubbing and washing testing, the PFSH sample linear resistivity values were, respectively, (37 Ω/cm) and (65 Ω/cm), and had the lowest resistivity compared to the other samples (Figure 12 and Figure 13). Similar resistance results were achieved by researchers of PANI-coated PET yarns ranging from 1.02 × 10^3^ Ω (PANI-spun 10 wt.%) to 1.53 × 10^6^ Ω (PANI-spun 3 wt.%) for a 10 cm long fiber [56,57]. In this report, a lower resistance of wool sample (PFSH) in comparison to the in situ polymerization PPy coated fabrics was obtained using the Tubicoat fixing agent HT and plasma treatment [55,56].

### 3.5. Spectrophotometric Determination of Color Differences

**Figure 14:**
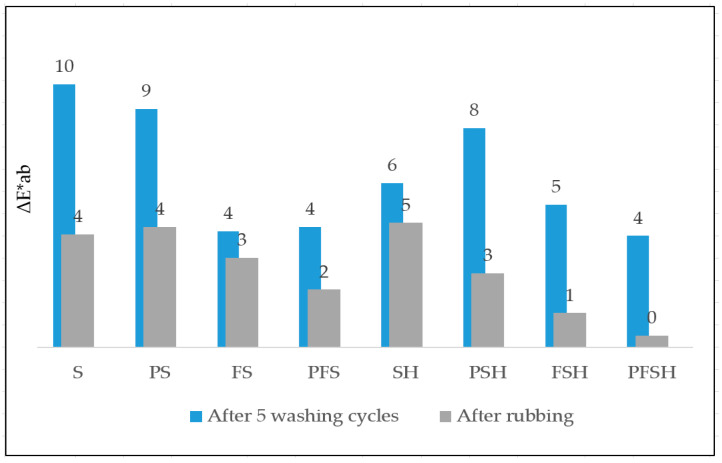
Color difference (ΔE*ab) test results after washing and rubbing testing with PEDOT: PSS-modified wool fabric samples by various methods.

Samples (S, PS, FS, PFS, SH, PSH, FSH, PFSH) of the wool fabric of different modifications references were compared with the same samples after washing and rubbing tests and performing color differences tests with a spectrophotometer. The PFSH sample has the least color difference (ΔE*ab) before and after washing and rubbing, resulting in the best resistance to washing and rubbing treatments. Samples (PS, PFS, PSH, PFSH) after plasma modification compared to unmodified plasma (S, FS, SH, FSH) showed lower color differences (ΔE*ab) and better resistance to mechanical treatment and washing (Figure 14). Providing the complex modification of sample (FS) dyeing with Clevios F ET and coating Clevios S V3, a smaller color difference compared to samples (S) printed only with Clevios S V3 was obtained (Figure 14).

Table 5 displays the measured L*, a*, b*, C*, and h coordinates and K/S values at the maximum wavelength of 1100 nm. Color yield K/S shows a higher dyeing ability [58] to PEDOT:PSS of the tested wool fabric samples (Table 5). The received results showed that after plasma treatment, the values K/S of samples (PS, PFS, PSH, PFSH) were lower compared to the unmodified K/S samples (S, FS, SH, FSH), as well as after washing and rubbing tests (Table 5). Plasma modified sample (PFSH) compared with the unmodified (FSH) was darker in color (L* = 36), had bluer (h = 240), more saturated colors (C* = 7), and had greener (a* = −4) and bluer (b* = −6) shade (Table 5). After five washing cycles, sample PFSH became yellowish (b* = 4), reddish (a*= 2), had a saturated color (C* = 5), a darker (L* = 34) shade, and had higher dyeing ability (K/S = 36) (Table 5). The K/S value determines the difference between the color strength of the sample PFSH reference (K/S = 35) and of the same sample after the rubbing test (K/S = 37). After the rubbing test, the sample PFSH color coordinates did not change much and were: darker (L* = 38), bluer (h = 239), had the same saturated color (C* = 7), greener (a* = −4), and had a bluer (b* = −6) shade (Table 5). The received results showed that plasma treatment increased wool fiber’s ability to react with PEDOT:PSS. Furthermore, the Tubicoat fixing agent HT as cross-linker enhanced the sample’s (PFSH)) resistance to washing and rubbing treatments [42].

**Figure 15:**
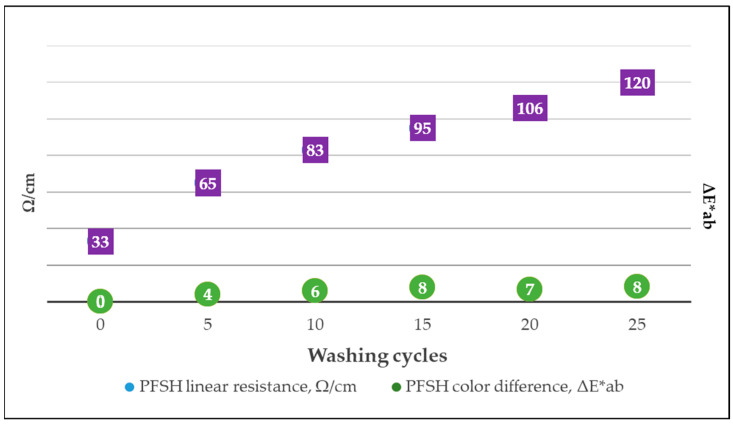
Linear resistance results with color difference comparison after 25 washing cycles of sample PFSH.

The sample PFSH with the best resistance to rubbing and washing was selected, and additionally, 25 washing cycles were carried out, measuring the linear resistance and color differences after every five washing cycles. The value of ∆E*ab shows that the magnitude of the color differences after the washing cycles is not very wide; after 15 washing cycles, the value remains almost constant at ∆E*ab of approximately eight. Moreover, the R_L_ of sample PFSH before washing was 33 Ω/cm (Figure 15). The R_L_ steadily increased to 120 Ω/cm when the 25 washing cycles were carried out, but the material was still electrically conductive (Figure 15) and could be classified as a dissipative material and used for protective workwear [59].

### 3.6. Fourier Transform Infrared Spectroscopy with Attenuated Total Internal Reflectance (FTIR-ATR) Mode Measurement

**Figure 16:**
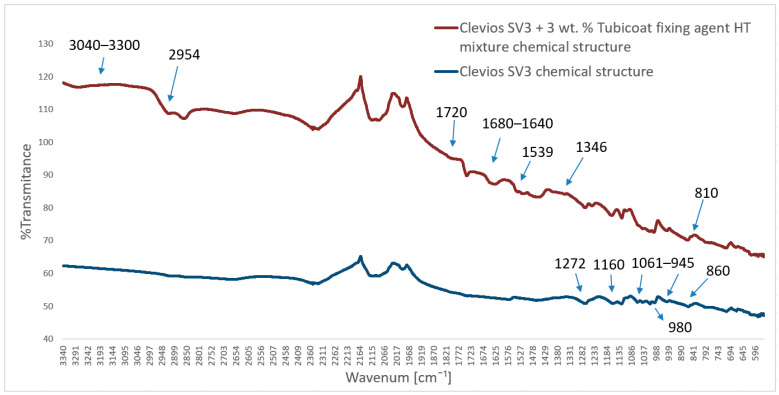
ATR infrared spectra of the Clevios S V3 with Tubicoat fixing agent HT and Clevios S V3 chemical structure.

FTIR-ATR spectra of the chemical structure of the pristine Clevios S V3 and composition of Clevios S V3 + 3 wt.% Tubicoat fixing agent HT spectra are presented in Figure 16. The principal absorption peaks of the PEDOT:PSS water dispersion in the form of Clevios S V3 were discovered at 1160 cm^−1^, corresponding to the symmetric and asymmetric stretch vibrations of the PSS sulfonic acid group, and the peaks were around 1030 cm^−1^, corresponding to the asymmetric stretching vibrations of benzene sulfonate [39,55,60]. The C–S bond stretching vibrations in the thiophene ring are responsible for the absorption peaks at approx. 860 and 980 cm^−1^ [61,62], as well as the C–O–C stretching at 1061 and 945 cm^−1^ and the C=C and C–C stretching in the quinoidal structure of PEDOT at 1272 cm^−1^ [35,63,64] (Figure 16).

IR spectra of sample Clevios S V3 + 3 wt.% Tubicoat fixing agent HT can be found in melamine resin from the band between 3040 and 3300 cm^−1^, with poor signal intensity, suggesting the potential O–H bond stretching vibration and N–H stretching vibration in primary aryl amines. These bands demonstrated that the cross-linking of formaldehyde and melamine was successful. The stretching vibration of C–H corresponds to the band of 2954 cm^−1^. Additionally, the methane vibration is represented by the new peak at roughly 1346 cm^−1^ [63,65]. The wavenumbers of amino-substituted triazines, which would exhibit a distinct absorption band in the range 1680–1640 cm^−1^, can be because of the reduced number of unreacted amino groups in the resin structure. The absorption band at 810 cm^−1^ corresponds to the out-of-plane bending vibrations of C–H bonds. Another clue that a tiny amount of aldehyde is still not engaged in the cross-linking process of melamine resin is the faint signal detected at 1720 cm^−1^ (a band characteristic for the C=O group) (Figure 16).

**Figure 17:**
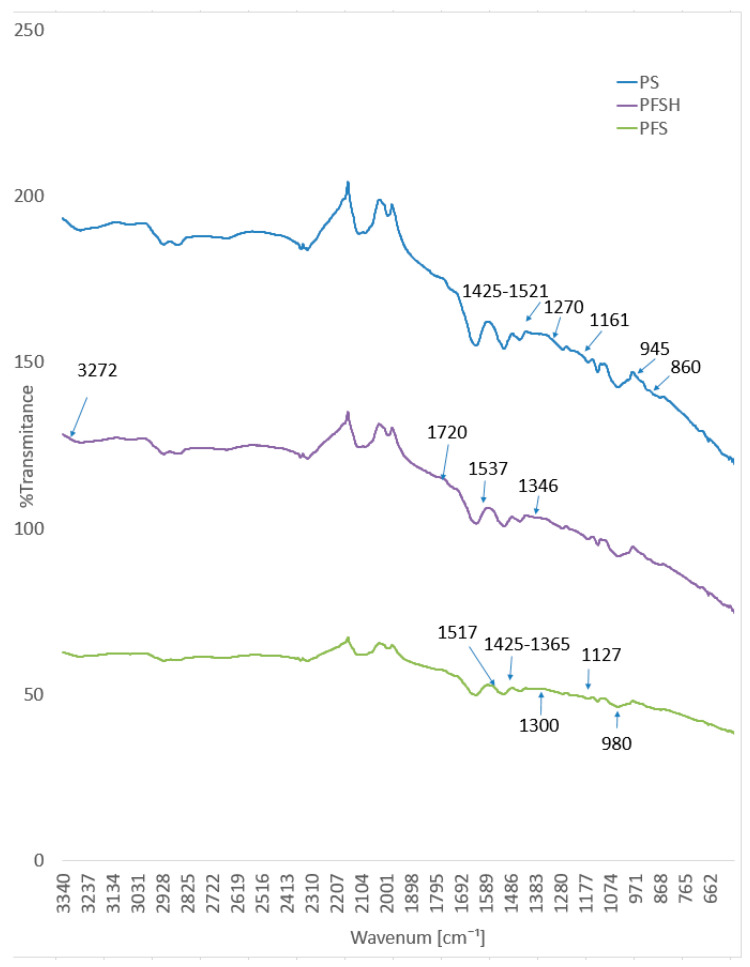
ATR infrared spectra of the wool samples PS, PFS, and PFSH.

FTIR-ATR infrared spectra of plasma treated samples: dyed with Clevios F ET and coated with Clevios S V3 (PFS) and Clevios F ET/Clevios S V3 + 3 wt.% Tubicoat fixing agent HT are shown in Figure 17. As described in our previous study [66], FTIR-ATR spectra of the wool surface after plasma treatment showed a decrease in aliphatic hydrocarbons (C–C, C–H), oxidation of –S–S–, an increase in the number of amino groups -N-H₂. This means that the N₂ plasma-treatment wool fabric samples have a more reactive and hydrophilic surface, resulting in better absorption of the PEDOT:PSS polymer [67].

All of the peaks from the wool fiber covered with PEDOT:PSS were nearly identical to the pure film of PEDOT:PSS dispersion (Figure 17). The principal peaks of the sample (PS) absorption were located at 1161 cm^−1^ (sulfonic acid group of PSS), 1065–945 cm^−1^ (C–O–C stretching), 945 cm^−1^ and 860 cm^−1^ (C–S stretching), and 1270 cm^−1^ (C=C and C–C stretching of the quinoidal structure of PEDOT) [64]. Specific wavelengths of specific PEDOT:PSS absorption peaks were found in Clevios F ET and Clevios S V3 spectra. Peaks at 1127 cm^−1^ (sulfonic acid group) and 1425 cm^−1^ (corresponding to the C=C and C–C stretching vibrations coming from the thiophene ring) were also found, showing that the PEDOT:PSS was successfully interpolated on the surface of the wool fabric (Figure 17). Similar PEDOT:PSS peaks were found by Mingwei Tian et al. on cotton fabric surfaces at 1517 cm^−1^ and 1300 cm^−1^ [35]. Similar spectra to Figure 16 are found on sample PFSH near 3272 cm^−1^ and 1537 cm^−1^, 1346 cm^−1^ wavelength range. These results, when combined with the signal at 1720 cm^−1^, which represents the stretching vibrations of the C=O, may indicate that there are still a few unreacted O–H molecules in the polymer and a small amount of aldehyde that is not yet involved in the cross-linking of melamine resin [44,45,63] (Figure 17).

## 4. Conclusions

The SEM images show that after plasma treatment dyeing with Clevios F ET and coating with Clevios S V3 3 wt.% Tubicoat fixing agent HT composition, the resulting coating is more rigid and evenly coated on the reverse side of the fabric. This may have improved rubbing and washing resistance. Using only coating with Clevios S V3, the surface coating is visible only on the surface. After plasma treatment, dyeing, and coating with PEDOT:PSS commercial products named Clevious, the linear resistivity was almost halved compared to coating with Clevios S V3 alone. The functional groups introduced onto the surface after N_2_ gas plasma treatment, and Tubicoat fixing agent HT of wool fabric were characterized by FTIR-ATR spectroscopy. The results of color difference measurement show that N_2_ plasma treatment and melamine formaldehyde Tubicoat fixing agent HT increased the resistance of PEDOT:PSS coated samples to washing and rubbing, and more intense color and electrical conductivity retaining were received.

## Figures and Tables

**Table 1:** Codification of samples processed by various methods.

Code of Sample	F	PF	S	PS	FS	PFS	SH	PSH	FSH	PFSH
Modifications	Clevios F ET	Plasma/Clevios F ET	Clevios S V3	Plasma/ Clevios S V3	Clevios F ET/ Clevios S V3	Plasma/Clevios F ET/ Clevios S V3	Clevios S V3 + Tubicoat fixing agent HT	Plasma/Clevios S V3 + Tubicoat fixing agent HT	Clevios F ET/ Clevios S V3 + Tubicoat fixing agent HT	Plasma/ Clevios F ET/Clevios S V3 + Tubicoat fixing agent HT

**Table 2:** Sample photo images before and after 5 washing 40 °C and drying cycles [48].

Code of Sample	F	PF	S	PS	FS	PFS	SH	FSH	PFSH
Before washing	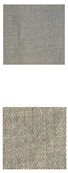	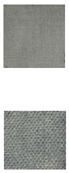	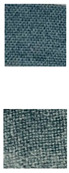	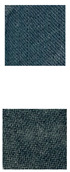	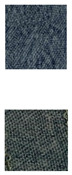	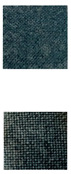	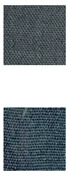	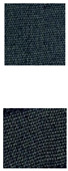	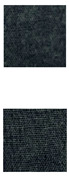
After 5 washing cycles									

**Table 3:** Wool samples with different modifications after rubbing tests [50].

Code of Sample	F	PF	S	PS	FS	PFS
Rubbing cloth	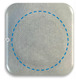	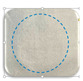	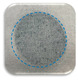	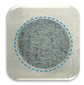	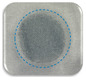	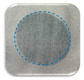

**Table 4:** Wool samples with different modifications with 3 wt.% of Tubicoat fixing agent HT after rubbing tests [50].

Code of Sample	SH	PSH	FSH	PFSH
Rubbing cloth	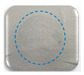	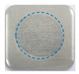	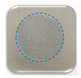	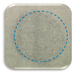

**Table 5:** Following washing and rubbing testing, we compared the L* a* b* C* h color coordinates and the maximal K/S values of unwashed plasma and PEDOT:PSS treated samples.

Code of Sample	Reference						After 5 Washes						After Rubbing Test					
	L*	a*	b*	C*	h	K/S	L*	a*	b*	C*	h	K/S	L*	a*	b*	C*	h	K/S
S	43	−3	−7	7	246	50	46	−2	−5	5	243	0.065	45	−3	−5	5	244	65
PS	38	−2	−7	7	251	36	44	−2	−5	5	245	0.071	41	−2	−6	6	250	51
FS	40	−3	−6	7	242	44	35	−2	−3	3	232	0.038	41	−3	−5	6	242	55
PFS	37	−4	−6	7	240	33	36	−2	−5	5	242	0.035	38	−3	−5	6	239	40
SH	40	−2	−6	7	250	43	48	−2	−5	5	245	0.079	46	−3	−4	5	239	73
PSH	38	−2	−7	7	252	39	45	−2	−5	6	247	0.065	38	−2	−7	7	252	34
PFSH	36	−4	−6	7	240	35	34	2	4	5	240	0.036	38	−3	−6	7	239	37

Note: (±0.1) confidence intervals.

## Data Availability

The data presented in this study are available on request from the corresponding author.
